# Systemic Concentrations of Short Chain Fatty Acids Are Elevated in Salmonellosis and Exacerbation of Familial Mediterranean Fever

**DOI:** 10.3389/fmicb.2016.00776

**Published:** 2016-05-24

**Authors:** Zhanna A. Ktsoyan, Mkhitar S. Mkrtchyan, Magdalina K. Zakharyan, Armine A. Mnatsakanyan, Karine A. Arakelova, Zaruhi U. Gevorgyan, Anahit M. Sedrakyan, Alvard I. Hovhannisyan, Arsen A. Arakelyan, Rustam I. Aminov

**Affiliations:** ^1^Institute of Molecular Biology of National Academy of Sciences of Republic of ArmeniaYerevan, Armenia; ^2^Clinical Hospital of Infectious Diseases Nork, Ministry of Health of Republic of ArmeniaYerevan, Armenia; ^3^School of Medicine and Dentistry, University of AberdeenAberdeen, UK

**Keywords:** *Salmonella*, salmonellosis, short chain fatty acids, inflammation, familial Mediterranean fever, peripheral blood

## Abstract

Gut microbiota-produced short chain fatty acids (SCFAs) play an important role in the normal human metabolism and physiology. Although the gradients of SCFAs from the large intestine, where they are largely produced, to the peripheral blood as well as the main routes of SCFA metabolism by different organs are known well for the healthy state, there is a paucity of information regarding how these are affected in disease. In particular, how the inflammation caused by infection or autoinflammatory disease affect the concentration of SCFAs in the peripheral venous blood. In this work, we revealed that diseases caused either by infectious agents (two *Salmonella enterica* serovars, *S.* Enteritidis, and *S.* Typhimurium) or by the exacerbation of an autoinflammatory disease, familial Mediterranean fever (FMF), both result in a significantly elevated systemic concentration of SCFAs. In the case of salmonellosis the concentration of SCFAs in peripheral blood was significantly and consistently higher, from 5- to 20-fold, compared to control. In the case of FMF, however, a significant increase of SCFAs in the peripheral venous blood was detected only in the acute phase of the disease, with a lesser impact in remission. It seems counterintuitive that the dysbiotic conditions, with a reduced number of gut microorganisms, produce such an effect. This phenomenon, however, must be appraised within the context of how the inflammatory diseases affect the normal physiology. We discuss a number of factors that may contribute to the “leak” and persistence of gut-produced SCFAs into the systemic circulation in infectious and autoinflammatory diseases.

## Introduction

Gut microbiota plays a fundamental role in the development and maintenance of many functions of the human body throughout life. The interactions with the host involve a number of metabolic, signaling, developmental, and immune processes. It is also important for the maintenance of integrity of the gut ecosystem and also for the protection against the invading pathogens (colonization resistance; Nicholson et al., 2012) via microbial and immune mechanisms (Stecher et al., 2010). Alterations in the composition and metabolic activities of the gut microbiota contribute to many chronic and degenerative diseases (Hawrelak and Myers, 2004). The interplay between the environmental factors such as antibiotic usage, diet, stress, infections and injury as well as the host genetic factors continuously shape the structure-and-function of the intestinal microbiota, with the consequences for human health. A number of dysbiotic conditions are associated with a wide spectrum of human diseases such as inflammatory bowel disease (IBD), colorectal cancer, irritable bowel syndrome (IBS), stomach ulcers and cancer, asthma, atopy, metabolic syndrome, diabetes, hypertension, autism, and other pathologies (Penders et al., 2007; Prakash et al., 2011; Petersen and Round, 2014; Parekh et al., 2015).

The interplay between the gut microbiota and immune system is complex. Although, it is well-established that the gut microbiota is essential for the proper development and maintenance of the immune system (Cerf-Bensussan and Gaboriau-Routhiau, 2010), there are indications that the immune system, on its turn, may also affect the composition of the gut microbiota (Khachatryan et al., 2008; Manukyan et al., 2008b). This interaction may have important implications for the development of inflammatory diseases, including autoimmune diseases and allergy, and the mechanisms by which the gut commensals drive the development of different types of immune responses are beginning to be understood (Licciardi et al., 2010).

Inflammation is the integral part of the normal innate immune response and acts as an early step in the cascade of reactions eventually resulting in the resolution of inflammation and healing (Headland and Norling, 2015). The sequence of events from the induction to resolution of inflammation in the course of infection of bacterial, viral, or protozoal etiology is fairly well-known. Nevertheless, some details of this process, in particular how the innate inflammatory response affects specific pathogens, commensal microbiota and the metabolome are less understood.

Regarding the inflammatory response, it becomes clear that it is not always detrimental to the pathogen. Some pathogens, for example, may use it for their own advantage during infection (Stecher et al., 2010). Activating the host immune defenses can suppress the protective commensal microbiota and facilitate the invasion and establishment of pathogens. *Salmonella*-induced inflammation, for example, results in the altered microbiota, which favors the growth of the pathogen (Barman et al., 2008). The ability of *Salmonella* spp. to overcome colonization resistance and successfully invade the host cells is partially due to its ability to utilize the inflammation-induced ecological and metabolic breaches to enhance its pathogenic potential (Winter et al., 2010; Ahmer and Gunn, 2011).

The normal intestinal microbiota generates high concentrations of short chain fatty acids (SCFAs) as the end products of anaerobic metabolism in the gut. The SCFAs produced execute multiple effects on the host as well as on the microbiota itself. Some are partially metabolized by the colonocytes (Velázquez et al., 1997) while the remaining SCFAs are cleared in the liver and then enter systemic circulation (Schuijt et al., 2012). The gradient of concentrations of the three main SCFAs (acetate, propionate, and butyrate) in the human body has been initially studied by Cummings et al. (1987). They found that the highest concentration of these three SCFAs is in the large intestine, with a gradual decrease in the portal, hepatic and peripheral venous blood. The concentration in peripheral blood was 1/1000th of that in the large intestine. The SCFAs delivered to various tissues then affect lipid, glucose, and cholesterol metabolism (den Besten et al., 2013). For example, SCFAs have a profound effect on the appetite regulation and energy homeostasis of the host (Byrne et al., 2015). They also contribute to the maintenance of immune homeostasis in the intestine and have multiple effects on the host cells involved in the inflammatory and immune pathways (Honda and Littman, 2012). In particular, SCFAs have a profound effect on regulation of T cells and contribute, directly or indirectly, to their differentiation (Kim et al., 2014). Other SCFAs effects on host physiology and metabolism include the regulation of epithelial cell growth and contribution to the functioning of the central and peripheral nervous system (Bienenstock et al., 2015).

Although it is known that SCFAs can modulate the function of immune cells, more studies are necessary in order to understand the precise role of specific gut bacterial SCFAs on host immune cells *in vivo* (Vinolo et al., 2011). The previous studies have been essentially limited to one of the main SCFAs produced, butyrate, and the experimental basis has been limited to animal models, *in vitro* experiments, and some clinical intervention studies. There are very few *in vivo* studies aimed at clarification of the SCFAs role in disease. For example, it has been demonstrated that individual SCFAs or their combination may enhance disease resistance and modulate the expression of an antimicrobial host defense peptide (Sunkara et al., 2011, 2012). High systemic concentrations of propionate and butyrate, however, are toxic and may impose adverse effects on the host (Bloemen et al., 2010). Still, as has been stated in a recent review by den Besten et al. (2013): “A coherent understanding of the multilevel network in which SCFAs exert their effects is hampered by the lack of quantitative data on actual fluxes of SCFAs and metabolic processes regulated by SCFAs.”

On the microbial side, the effects of SCFAs are mainly metabolic, supporting a number of syntrophic relationships in the gut (Pimentel et al., 2012). At the same time, in some pathological inflammatory conditions such as infection these substances can be used by a pathogen to enhance its virulence potential for the invasion of the intestinal epithelium of the host (Winter et al., 2010).

In our previous works we have established that in diseases involving an inflammatory component the concentrations and profiles of gut microbial products in systemic circulation, in particular that of long chain fatty acids, are substantially different compared to control (Ktsoyan et al., 2011, 2013a). The main aim of the present study was to establish how the quantity and proportion of the SCFAs, which are generated by the commensal microbiota and then enter systemic circulation, are affected in two diseases. The first disease investigated included infections by two *Salmonella enterica* serovars, and the second – an autoinflammatory disease, familial Mediterranean fever (FMF; Brydges and Kastner, 2006; Manukyan and Aminov, 2016). In both conditions we observed the increase of systemic concentrations of SCFAs as well as changes in the molar ratio of main SCFAs.

## Materials and Methods

In this study, which was performed over a 2-years period from 2013 to 2015, we examined 64 Armenians residing in the territory of Republic of Armenia. Five cohorts were investigated: (i) control group of healthy subjects (*n* = 18); (ii) patients with acute salmonellosis caused by *S.* Typhimurium (*n* = 11) or (iii) *S.* Enteritidis (*n* = 15) infections; (iv) FMF patients with acute inflammation (*n* = 9); and (v) FMF patients in remission (*n* = 11).

All patients and controls belonged to the same ethnic group. The FMF cases were diagnosed based on Tel-Hashomer criteria with further genetic confirmation of *MEFV* mutations (Livneh et al., 1997). None of the FMF patients and healthy individuals used antibiotics within 3 months prior to sampling.

The cohorts of salmonellosis patients included subjects admitted to the infectious disease hospital *Nork* in Yerevan, RA. Diagnoses were based on clinical presentations and laboratory analyses. Clinical appearances consistent with gastroenteritis were diarrhea, fever, nausea, vomiting, and abdominal cramps. Anamnesis included food and water consumed, social gatherings, relatives and friends with the similar symptoms, and the history of any recent travel.

The salmonellosis patient selection criteria for this study included not taking any type of medication including antibiotics before the hospital admission. Blood and fecal samples were taken on the first or the second day of hospital admission. At the time of discharge from the hospital, no *Salmonella* presence was detected in the feces or blood of the patients.

Biochemical tests for the identification of *Salmonella* were fermentation of glucose, negative urease reaction, lysine decarboxylase, negative indole test, H_2_S production, and fermentation of galactitol (dulcitol). Serotypes of *Salmonella* were determined using the standard Kauffman–White scheme with the use of commercially available polyvalent antisera for flagellar (H) and lipopolysaccharide (O) antigens.

All subjects in the study, or their parent or guardian if a child, were informed about the aim of this study and gave their written consent to participate in it. The study protocol was approved by the Ethics Committee of the Institute of Molecular Biology NAS RA (IORG number 0003427, Assurance number FWA00015042, and IRB number 00004079).

Quantitative and qualitative determination of SCFAs in the peripheral blood of patients was performed by gas chromatography (GC) using Varian GC CP–3380 (Varian, Inc., USA) and WCOT Fused Silica type columns (length – 50 m and inner diameter – 0.32 mm). The blood samples were taken by venipuncture in the amount of 5 ml. After the clot formation, a sample was centrifuged and pH of 1 ml of supernatant was adjusted to 2.0 with 50% sulfuric acid. The extraction with 1 ml of diethyl ether was performed twice and then evaporated down to 100 μl. A portion of the resulting ether extract (1 μl) was subjected to GC analysis. Identification of SCFAs was performed by determining the relative retention times; commercially available analytical grade SCFAs were used as standards. Quantification of SCFAs was done by comparing the height of peaks to the standard solutions with known concentrations. Before each series of tests the steady state of chromatograph operation was achieved, followed by calibration with standards.

GraphPad Prism 5 (GraphPad Software, USA) was used to perform the Mann–Whitney *U*-test to determine the statistical significance of differences among the groups studied. The *p*-values < 0.05 were considered statistically significant. Discriminant function analysis was carried out with the IBM SPSS Statistics 19 package (IBM, USA).

## Results

### Systemic SCFAs in Salmonellosis Patients

**FIGURE 1:**
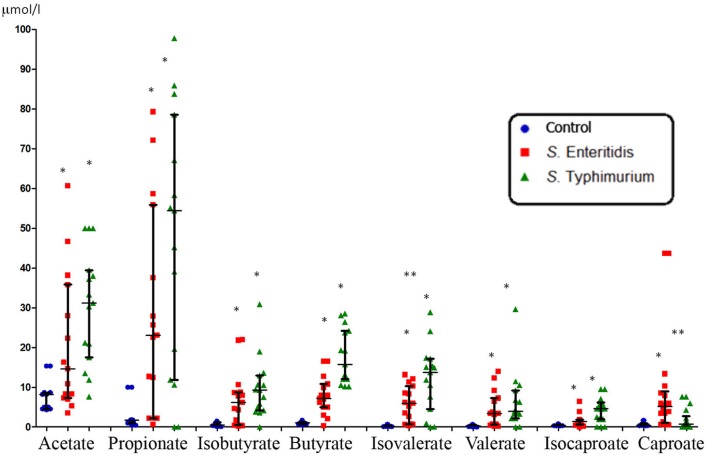
**Systemic concentration of SCFAs in patients infected with two serotypes of *Salmonella enterica* (median with interquartile range).** Statistically significant differences in SCFA concentrations: ^∗^ significant difference between infected and control subjects (*p* ≤ 0.05, Mann–Whitney *U*-test) and ^∗∗^ significant difference between *S*. Enteritidis- and *S*. Typhimurium-infected patients (*p* ≤ 0.05, Mann–Whitney *U*-test).

The concentration of SCFAs in the peripheral blood of healthy subjects was found to be consistently low (**Figures 1** and **3**). This is not surprising since it is well-known that these microbial metabolites are actively metabolized, both *in situ* in the gastrointestinal tract (GIT) and in other organs such as the liver, muscles and other organs and tissues. On the contrary, the concentration of SCFAs in the peripheral blood of patients with acute salmonellosis caused by *S*. Enteritidis and *S*. Typhimurium infections was significantly and consistently higher compared to control (from 5- to 20-fold, *p* < 0.05; **Figure 1**). The only exception was the concentration of caproate, which was significantly increased only in patients infected by *S*. Enteritidis, but not by *S*. Typhimurium, compared to control. Between the two serovars, significant differences (*p* < 0.05) were detected in the concentration of isovalerate (higher in *S*. Typhimurium) and caproate (higher in *S*. Enteritidis; **Figure 1**).

**FIGURE 2:**
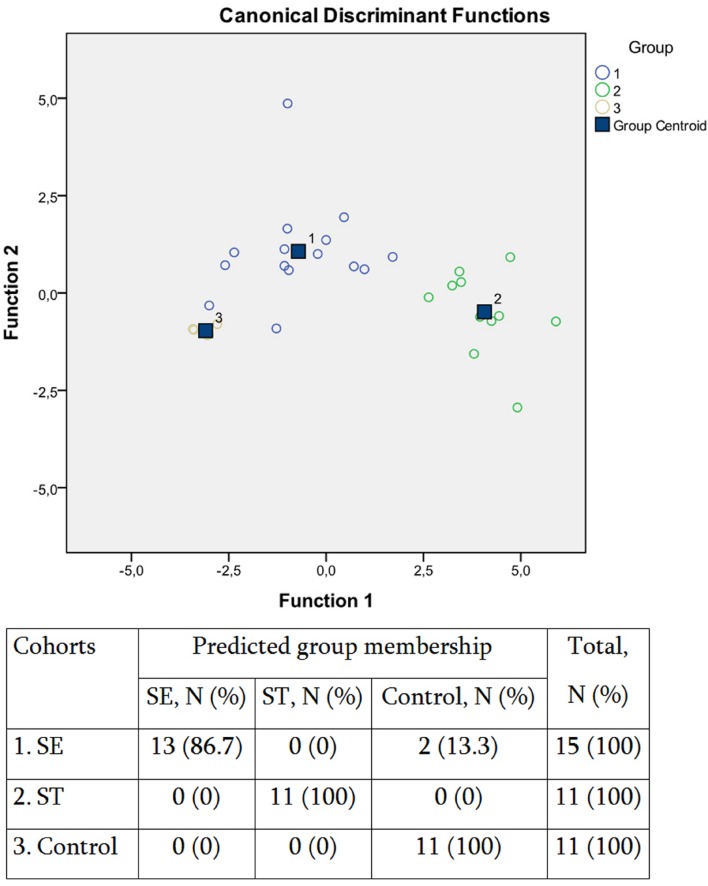
**Scatterplot of DA model and the predicted group membership in the model based on systemic concentration of microbial SCFAs in salmonellosis patients and control subjects.** Number of variables in the model – 8 and grouping consists of three groups. Root 1, 2 – discriminant functions 1 and 2 (1st and 2nd canonical roots). Cohorts: (1) *S*. Enteritidis-infected patients (SE), (2) *S*. Typhimurium-infected patients (ST), (3) control subjects. Predictive accuracy of classification: 94.7% of the original group cases were correctly classified; Wilks’ λ = 0.055.

The SCFA concentration data were further subjected to multivariate statistic analyses, namely discriminant function analyses (DA; **Figure 2**). The predictive accuracy of model based on the SCFA concentration variables was sufficiently accurate at 94.7%, with only two *S*. Enteritidis infection cases being allocated to the control group (**Figure 2**). These results suggested that despite the similarity in the increase of SCFAs concentrations in response to both *S. enterica* infections, there is certain specificity in the corresponding SCFA profile, which depends on the serotype of the pathogen. We have found earlier that the inflammatory response in salmonellosis is serotype-specific (Ktsoyan et al., 2013b, 2015). The differential immune response probably results in the differential composition of the gut microbiota, with the subsequent distinctive profile of SCFAs excreted.

### Systemic SCFAs in Autoinflammatory Disease FMF

**FIGURE 3:**
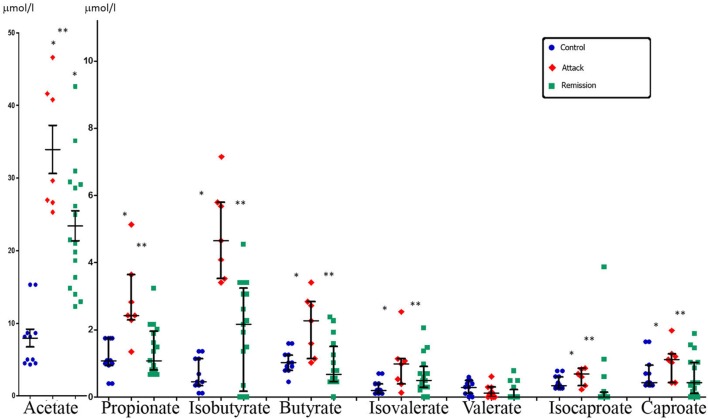
**Systemic concentration of SCFAs in FMF patients in attack and remission periods and in control subjects (median with interquartile range).** Statistically significant differences in SCFA concentrations: ^∗^ significant difference between FMF attack and control (*p* ≤ 0.05, Mann–Whitney *U*-test) and ^∗∗^ significant difference between FMF attack and remission (*p* ≤ 0.05, Mann–Whitney *U*-test). The concentration of acetate is shown in a different scale.

Systemic concentration of the majority of SCFAs in FMF patients during disease exacerbation was consistently higher compared to control (**Figure 3**). Statistically significant differences were detected for almost all SCFAs, except valerate. In remission, however, the levels of SCFAs were much lower compared to the attack period and were closer to the values in the control group, except acetate (**Figure 3**). Comparison between the disease stages revealed that the concentration of the majority of SCFAs was significantly higher during the attack period compared to remission except for valerate (**Figure 3**).

**FIGURE 4:**
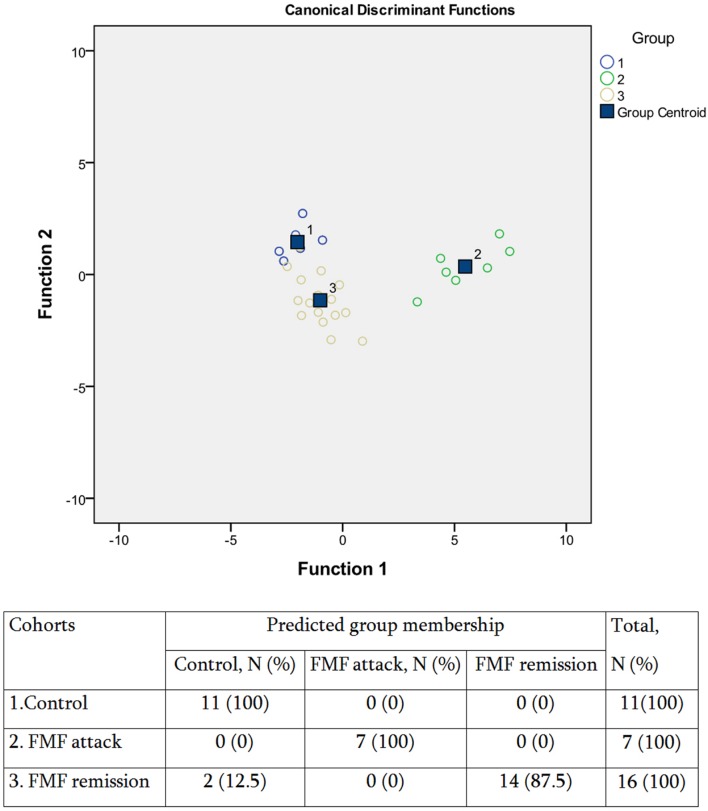
**Scatterplot of DA model and the predicted group membership in the model based on systemic concentration of microbial SCFAs in FMF patients (attack and remission) and in control subjects.** Number of variables in the model – 8 and grouping consists of three cohorts. Root 1, 2 – discriminant functions 1 and 2 (1st and 2nd canonical roots). Groups: (1) control, (2) FMF attack, and (3) FMF remission. 0.94.1% of the original group cases were correctly classified; Wilks’s λ = 0.042.

Consistently with these observations, our further DA analysis of the three groups revealed a closer clustering of the control and FMF remission groups compared to the disease exacerbation group (**Figure 4**). In general, however, the model had a good predictive power, with an overall 94.1% accuracy of classifications. Thus, despite that the systemic concentration of the majority of SCFAs is not significantly different between the FMF remission and control groups, there is still a specific profile of SCFAs in disease remission allowing to separate this group from control. We have shown earlier that although FMF remission is characterized by the absence of clinical signs of disease, the elevated level of cytokines and CRP suggest the persisting subclinical inflammation (Manukyan et al., 2008a). Thus the subclinical inflammation during the remission periods may affect the composition of gut microbiota and, subsequently, the specific profile of SCFAs excreted.

Thus the results demonstrated that the systemic concentration of microbially produced SCFAs in acute FMF is significantly elevated compared to control. The concentration of the majority of SCFAs in FMF remission is not significantly increased compared to control but, nevertheless, displays a SCFAs profile different from that of control.

### Ratio of the Main Systemic SCFAs

Finally, we determined the molar ratio of the main SCFAs in peripheral venous blood (**Table 1**). The three most abundant SCFAs generated by gut microbiota are acetate, propionate and butyrate, together comprising more than 95% of all SCFAs (Cook and Sellin, 1998), and which are present in a molar ratio of about 60:20:20 in the large intestine (Cummings et al., 1987). In the peripheral blood these three SCFAs also remain dominant. In our experiments we also found that these SCFAs are dominant in the venous blood of all patients and controls (**Figures 1** and **3**). The molar ratio of acetate:propionate:butyrate in healthy subjects was approximately 80:10:10 (**Table 1**) suggesting the preferential utilization of the two latter SCFAs. In salmonellosis the proportions of butyrate and propionate increased up to 15 and 50%, correspondingly, at the expense of acetate, which proportion fell dramatically to 35% (**Table 1**). On the contrary, the proportion of systemic acetate in FMF is even higher compared to control (90 vs. 80%), with the proportional decrease of the percentage of propionate and butyrate, both falling to 5% (**Table 1**).

**Table 1:** Molar ratio of three main systemic SCFAs in salmonellosis and FMF patients.

Cohort	Acetate (%)	Propionate (%)	Butyrate (%)
Control	78.19 ± 37.9	11.49 ± 4.8	10.32 ± 3.14
*S*. Enteritidis	35.62 ± 30.11	50.63 ± 44.3	13.75 ± 8.1
*S*. Typhimurium	31.72 ± 14.5	49.64 ± 33.1	18.64 ± 7.0
FMF (attack)	87.14 ± 20.8	7.36 ± 2.9	5.5 ± 2.15
FMF (remission)	91.15 ± 32.1	4.925 ± 2.5	3.925 ± 3.0


## Discussion

In this work, we established that two types of diseases with an inflammatory component, of the infectious and autoinflammatory nature, result in the significantly elevated systemic levels of microbially produced SCFAs. In the autoinflammatory disease, FMF, this increase is mainly associated with the acute inflammation stage but even in remission the profile of SCFAs remains biased compared to control. To the best of our knowledge this is the first attempt to measure systemic concentrations of SCFAs in pathological conditions such as a gastrointestinal infection or an autoinflammatory disease.

Microbial anaerobic metabolism of dietary and endogenous substrates in the human gut results in the production of SCFAs and gasses such as hydrogen, methane, and carbon dioxide. A pioneering effort to establish the fate of the three main microbially produced SCFAs (i.e., acetate, propionate, and butyrate) has been made by Cummings et al. (1987) using the autopsy material of sudden death victims. They determined the SCFAs concentration in different parts of the large intestine and in portal, hepatic and peripheral venous blood and found that the concentration in peripheral blood was 1/1000th of that in the intestine. They also noticed a marked difference in the molar ratio of these SCFAs in the three parts of circulation. Our data in regards to the concentration of propionate and butyrate in the peripheral blood of healthy volunteers are consistent with these earlier findings. The concentration of acetate, however, is substantially lower possibly reflecting other confounding factors. Besides colonic fermentation, acetate can be produced endogenously (Skutches et al., 1979; Scheppach et al., 1991). The level of acetate is also affected by the host metabolism since its involvement in cholesterol biosynthesis and lipidogenesis, as a cosubstrate for glutamine and glutamate synthesis, as well as serving as energy source for many organs and tissues (Knowles et al., 1974; Wong et al., 2006; den Besten et al., 2013). A substantially lower concentration of acetate in peripheral venous blood compared to that obtained by Cummings et al. (1987) has been also reported by others (Dankert et al., 1981). The molar ratio of acetate to propionate to butyrate concentrations in the peripheral blood of healthy subjects, 80:10:10, is within the range of the previously published data (Cummings et al., 1987; Peters et al., 1992; Bloemen et al., 2009).

It has been demonstrated in the murine models of *S*. Typhimurium infection that it results in the disruption of the indigenous gut microbiota, with a drastic decrease in the total number of bacteria and alteration of the microbiota composition (Barman et al., 2008; Sekirov et al., 2010). In the porcine model of *S*. Typhimurium infection as well, a general reduction of gut microbiota and, in particular, of SCFA-producing bacteria, can be seen (Drumo et al., 2016). Previously, we have confirmed that in humans as well, salmonellosis causes a considerable degree of dysbiosis (Ktsoyan et al., 2015). In the dysbiotic condition accompanied by diarrhea, therefore, the concentration of SCFAs is expected to be decreased in the intestinal milieu, with the corresponding proportional decrease in systemic circulation.

However, the opposite trend was observed, with a significantly increased concentration of SCFAs in the peripheral venous blood of salmonellosis patients (**Figure 1**). The molar ratio of acetate:propionate:butyrate was also significantly affected, with a drastic decrease of the proportion of acetate and the increased proportions of propionate and butyrate (**Table 1**). The increase in concentration and proportion of butyrate in peripheral blood may be the consequence of intestinal inflammation and impaired function of colonocytes, for which butyrate is the preferred energy source (Velázquez et al., 1997). Thus the less efficient catabolism of butyrate by the inflamed gut tissue may lead to its elevated concentration in peripheral blood.

Propionate is largely metabolized by the liver serving as a precursor for gluconeogenesis (Roy et al., 2006). Severe enterocolitis due to *S*. Enteritidis infection affects liver enzyme levels (González-Quintela et al., 2004) suggesting that the liver function may be impaired in this condition. Because of the increased residual concentration of propionate in blood, the most likely affected function is its transport. In the hepatocytes, organic anion transporters OAT2 and OAT7 are responsible for the transport of propionate and butyrate, correspondingly, from blood, across the sinusoidal membrane (Shin et al., 2007; Islam et al., 2008). The impaired transport from blood to the hepatic tissue may, therefore, explain the insufficient clearance of propionate (and butyrate) by the liver leading to their elevated level in the peripheral blood of the salmonellosis patients. Under the normal circumstances there is a substantial amount of butyrate that leaves the GIT, and it is cleared by the liver (Bloemen et al., 2009). The normal hepatic uptake is capable of clearing even the elevated concentrations of artificially administered butyrate thus preventing the increase in systemic butyrate concentrations (van der Beek et al., 2015).

As mentioned before, acetate is the main SCFA produced by gut microbiota, and there is also a substantial endogenous production of it by the human body (Skutches et al., 1979; Scheppach et al., 1991). Acetate is also involved into a number of anabolic and catabolic biochemical pathways in different tissues and organs (Knowles et al., 1974; Wong et al., 2006; den Besten et al., 2013). It is difficult, therefore, to point out to a single factor that may explain the elevated systemic level of acetate in salmonellosis. Investigation of the uptake of ^11^C-acetate by different normal organs demonstrated that it is absorbed by many organs and, especially actively, by the pancreas, liver, spleen and salivary glands (Song et al., 2009). In the case of salmonellosis many organs can be affected including the liver and spleen. Spadoni et al. (2015) described a gut-vascular barrier, which prevents the intestinal bacteria entering the bloodstream and reaching the organs. But the pathogens such as *S*.Typhimurium can penetrate this barrier employing the pathogenicity island II-encoded type III secretion system and the decreased β-catenin-dependent signaling in the gut cells. This allows them to enter systemic circulation and reach the liver and spleen thus affecting the function of these organs, possibly including the uptake of acetate. We hypothesize that this scenario may lead to the observed increase of systemic concentration of acetate in salmonellosis patients. The molar ratio of acetate in systemic circulation, however, is substantially lower than that of propionate and, to a lesser extent, of butyrate compared to control (**Table 1**) thus suggesting that the main organ affected may be the liver where the bulk of propionate is cleared.

Regarding other minor SCFAs, their concentrations in the peripheral blood of salmonellosis patients are also significantly elevated (**Figure 1**). We cannot, however, compare our results on minor SCFAs with others because of the lack of the relevant publications. In summary, despite that the dysbiosis caused by salmonellosis affects SCFAs-producing bacteria (Drumo et al., 2016) and presumably the gut production of SCFAs as well, the concentrations of SCFAs in the peripheral blood are significantly elevated thus reflecting the failure of disease-affected organs such as the liver to clear SCFAs. This may be also relevant to other metabolites produced by gut microbiota.

In an autoinflammatory condition, FMF, the increase in systemic SCFAs is caused by a different set of factors. First of all, the increase is observed only in the active disease, while it remains close to the control levels in remission except acetate (**Figure 3**). Previously we have shown that the active FMF is characterized by dysbiosis, with a significant decrease in the total number of gut bacteria and a substantial restructuring of the gut microbiota composition (Khachatryan et al., 2008). In remission, however, these dysbiotic changes are less evident and the diversity values may even exceed that of controls. Thus, in agreement with our earlier observations, in FMF remission we do not see a substantial departure from control in terms of the concentrations of SCFAs in the peripheral blood (**Figure 3**). The only exception is acetate, the concentration of which is increased almost threefold compared to control. This may be a consequence of subclinical low-grade inflammation in FMF remission (Manukyan et al., 2008a), which may interfere with the normal and efficient uptake of acetate by different organs (Song et al., 2009). The molar ratio of acetate against two other major SCFAs, propionate and butyrate, is elevated compared to control (**Table 1**) supporting this notion.

In active FMF, though, the concentrations of almost all SCFAs (except valerate) in the peripheral blood are significantly increased (**Figure 3**). In this regard the condition resembles the aforementioned infectious disease, salmonellosis. The disease mechanisms, however, are different: FMF is a hereditary disease caused by mutations in the *MEFV* gene (The French FMF Consortium, 1997; The International FMF Consortium, 1997). Earlier we have suggested that the heightened sensitivity of the immune system conferred by these mutations leads to inappropriate immune responses, launched, in particular, against gut commensals (Manukyan et al., 2008b). The disease exacerbation results in fever and polyserositis, with 95% of patients experiencing abdominal pain resembling the signs of peritonitis or appendicitis (Livneh and Langevitz, 2000). Despite the different cause of inflammation in FMF attack and salmonellosis, the consequence of both inflammatory conditions is the elevated level of gut-produced SCFAs in systemic circulation (**Figures 1** and **3**). The enhanced “leak” of microbially produced substances such as hydroxy, branched, cyclopropyl and unsaturated fatty acids, aldehydes, and phenyl derivatives into systemic circulation in FMF has also been noticed by us previously (Ktsoyan et al., 2011, 2013a). Together with the SCFAs data, the presence of various types of microbial products in systemic circulation may suggest a less efficient clearance of these compounds by the liver in these patients. Contrary to salmonellosis, however, the molar ratio of acetate:propionate:butyrate is not significantly affected in FMF patients in remission or attack, although there is a tendency for the increased acetate percentage, with the corresponding decrease in the proportion of propionate and butyrate (**Table 1**).

In summary, our results suggest that two dissimilar diseases, of infectious and autoinflammatory nature, both result in the elevated level of gut microbiota-produced SCFAs in systemic circulation. Both these disease, however, share a common component, i.e., active inflammation. We hypothesize that the inflammation leads to two major consequences. First, to the compromised gut barrier function (Bischoff et al., 2014), which then results in excessive translation of the luminal content, including SCFAs, into systemic circulation. And second, inflammation affects the intestinal epithelium and the organs beyond the GIT such as the liver that are involved in metabolism and clearance of microbial metabolites and compounds. These hypotheses, however, need further experimental verification.

## Author Contributions

AM and ZG performed sample collection; MZ, KA, AS, and AH performed experiments; AA and MM performed statistical analyses; RA and ZK performed data analyses, interpretation of data, and writing the paper.

## Conflict of Interest Statement

The authors declare that the research was conducted in the absence of any commercial or financial relationships that could be construed as a potential conflict of interest.
